# Cross‐Institutional Five‐Class Kellgren–Lawrence Grading of Knee Osteoarthritis via Multitask Deep Learning

**DOI:** 10.1111/nyas.70254

**Published:** 2026-03-14

**Authors:** Tariq Alkhatatbeh, Ahmad Alkhatatbeh, Yan Liao, Zhilin Zhang, Hang Fang, Weidong Chen, Rongkai Zhang

**Affiliations:** ^1^ Department of Joint Surgery, Center for Orthopaedic Surgery The Third Affiliated Hospital of Southern Medical University (Academy of Orthopedics Guangdong Province) Guangzhou China; ^2^ Orthopedic Hospital of Guangdong Province Guangzhou China; ^3^ Guangdong Provincial Key Laboratory of Bone and Joint Degeneration Diseases Guangzhou China; ^4^ Department of Orthopedics The First Affiliated Hospital of Shantou University Medical College Shantou City China

**Keywords:** deep learning, domain shift, Kellgren–Lawrence (KL), knee osteoarthritis (KOA), multitask architecture

## Abstract

Deep learning models for Kellgren–Lawrence (KL) grading often report optimistic performance due to data leakage and fail to generalize across institutions because of domain shift. To address this reproducibility crisis, we introduce KL‐FuseNet, a multitask architecture fusing global (ConvNeXt‐Base) and local (ResNet‐50) features to predict ordinal grades, label distributions, and binary severity (KL≥2). Using strict patient‐wise stratified splits on an internal osteoarthritis initiative dataset (*n* = 8260) and an independent Chinese cohort (*n* = 2295), we compared zero‐shot transfer against selective fine‐tuning. KL‐FuseNet achieved robust internal agreement (quadratic Cohen's kappa [QWK]: 0.881; accuracy: 70.3%). While external zero‐shot deployment revealed a domain gap, with accuracy dropping to 66.1%, our selective fine‐tuning protocol significantly bridged this divide, boosting external accuracy to 80.0% and QWK to 0.950, with an AUC of 0.984 for clinically significant osteoarthritis (KL≥2). These results demonstrate that while KL‐FuseNet achieves state‐of‐the‐art performance under rigorous evaluation, domain‐aware adaptation is essential for clinical utility. This study establishes a reproducible pathway for deploying automated grading models across heterogeneous medical centers.

## Graphical abstract

KL‐FuseNet performs automated Kellgren–Lawrence grading from single‐view knee radiographs by fusing one global stream with two shared‐weight bilateral patch streams. Trained under leakage‐safe patient‐wise splitting, the model uses ordinal and label‐distribution supervision with validation‐based early stopping and test‐time augmentation. It shows strong internal agreement and robust cross‐institution transfer after fine‐tuning, supporting reproducible, clinically oriented knee osteoarthritis (OA) severity assessment.

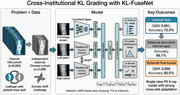

## 1 Introduction

Knee osteoarthritis (KOA) is the most common form of arthritis and a major contributor to global disability, with an estimated 374.74 million prevalent cases worldwide in 2021, representing an age‐standardized prevalence rate of 4294.27 per 100,000 population, alongside 30.85 million incident cases and 12.02 million disability‐adjusted life years [1]. Plain radiography remains the primary imaging modality for diagnosing and monitoring KOA, with the Kellgren–Lawrence (KL) grading system serving as the gold standard for assessing severity on a 5‐point ordinal scale based on features such as joint space narrowing, osteophyte formation, and subchondral sclerosis [2]. However, manual KL grading is highly subjective, with intrarater reliability ranging from 0.26 to 0.88 and inter‐rater reliability values from 0.56 to 0.80 across studies, leading to inconsistencies that particularly affect adjacent grades and undermine clinical and research outcomes [3, 4].

Deep learning has been widely adopted for automating KL grading, providing objective and reproducible assessments by capturing both global joint alignment and local pathological features [5]. Early models treated the task as standard multiclass classification using architectures like visual geometry group (VGG) or ResNet, but often overlooked the ordinal nature of KL grades [6]. More recent advancements have incorporated multistream designs, ordinal losses, and label‐distribution modeling to handle grade ambiguity [7]. Recent studies have also explored data augmentation and modern training pipelines for knee OA radiograph classification and automated KL grading [8, 9]. Despite these innovations, many studies do not enforce strict patient‐wise splits, which can lead to optimistic performance estimates when correlated images from the same participant (e.g., bilateral knees or repeated exams) are separated across training and evaluation partitions. In such settings, correlated subject‐specific cues can be shared across partitions, inflating apparent accuracy and quadratic Cohen's kappa (QWK). This issue is compounded by class imbalance in datasets, where extreme grades (KL0 and KL4) dominate performance, further masking weaknesses in challenging intermediate grades like KL1 [10].

Recent models have advanced from basic classification to sophisticated multitask frameworks, emphasizing robust baselines and improved early grade detection. Initial Siamese convolutional neural networks (CNNs) achieved QWK 0.83 on internal data but lacked rigorous external validation [5]. Lightweight approaches like EfficientNet‐B0 with efficient channel attention reached 92% binary accuracy (KL0 vs. KL1) while outperforming baselines such as VGG‐16 (85% accuracy) and ResNet‐50 (88% accuracy) [11]. Ensemble networks fusing DenseNet‐161 and ResNet‐101 attained 76.93% accuracy and 0.7693 macro‐F‐1 score, surpassing prior works like OsteoHRNet (71.74% accuracy) and Tiulpin et al. (74.0% accuracy) on imbalanced data [12]. Other CNN variants achieved 92.3% binary and 78.4% multiclass accuracy, exceeding traditional machine learning baselines [13], while plug‐in modules improved KL1 detection to 43% on independent sets [14]. Yet, many studies still report inflated internal metrics without addressing patient‐wise splits, as non‐region of interest (ROI) models can paradoxically boost performance for ambiguous grades by leveraging extraneous features [8].

Beyond internal validation, the clinical deployment of deep learning models for KL grading faces significant challenges from domain shifts arising from differences in scanner manufacturers, acquisition protocols, image quality, and patient demographics across institutions. Cross‐site generalization failures are well‐documented in medical imaging: Zech et al. demonstrated that a pneumonia detection model achieving AUC 0.93 on internal chest radiographs dropped to AUC 0.73 on external data, but recovered to AUC 0.87 after fine‐tuning with target‐site examples [15]. Similarly, Gulshan et al. showed that diabetic retinopathy models required fine‐tuning on external datasets to maintain clinical‐grade sensitivity across different fundus camera systems [16]. In KOA specifically, Tiulpin et al. trained on the MOST cohort and achieved QWK ∼0.83 on external osteoarthritis initiative (OAI) data, with subsequent fine‐tuning improving cross‐dataset performance [5], yet systematic quantification of the zero‐shot versus fine‐tuned performance gap under rigorous patient‐wise evaluation remains limited. A comprehensive methodological framework for medical imaging transfer learning by Tajbakhsh et al. established that selective fine‐tuning on modest target‐domain datasets consistently outperforms zero‐shot transfer across diverse imaging tasks [17], supporting domain adaptation as a necessary strategy for multisite clinical artificial intelligence (AI) deployment.

The complex nature of KOA assessment requires models that capture both global joint alignment and localized pathological features, such as osteophytes and joint space narrowing. The ordinal structure of the KL scale and ambiguity between adjacent grades, especially KL0 and KL1, demand strategies beyond standard multiclass classification.

These factors motivate our multistream, multitask architecture, which fuses global and local radiographic views via dual backbones (ConvNeXt‐Base and shared ResNet‐50) and incorporates specialized heads for ordinal ranking, label‐distribution ambiguity modeling, and binary detection of clinically significant osteoarthritis (KL ≥2). Critically, we evaluate our model under three distinct scenarios: (1) internal validation with strict patient‐wise splits on the OAI cohort; (2) zero‐shot external transfer to an independent Chinese cohort acquired on different equipment; and (3) selective fine‐tuning on external training data followed by held‐out test evaluation. This protocol enables us to quantify the domain gap, demonstrate the necessity of adaptation, and deliver realistic performance estimates that address key methodological pitfalls in the field.

## 2 Methods

### 2.1 Datasets and Splitting

Posteroanterior knee radiographs were sourced from two cohorts. The internal OAI subdataset included 8260 images from 4130 unique patients with consensus KL grades (0–4), obtained from a public, deidentified Mendeley Dataset [18]. Images follow the naming convention {patient_ID}{L|R}, where the last character denotes laterality (e.g., 9001400L and 9001400R represent bilateral knees from patient 9001400). Patient‐wise splitting was implemented by: (1) extracting unique patient IDs by removing the terminal L/R character; (2) computing each patient's maximum KL grade across bilateral images; (3) applying stratified splitting on the patient level (seed = 42) with 70/15/15 allocation; and (4) assigning all images from each patient to the corresponding partition. This yielded 2891 patients (5782 images) for training, 620 patients (1239 images) for validation, and 619 patients (1239 images) for testing, with guaranteed zero patient overlap. We emphasize this participant‐level (“patient‐wise”) split as a key methodological contribution for reproducible KL‐grading evaluation, because it prevents bilateral leakage (paired L/R knees from the same participant appearing in different partitions). Table 1 summarizes the split proportions and counts.

**TABLE 1:** Summary of datasets and class distributions.

Cohort	Count	KL 0	KL 1	KL 2	KL 3	KL 4
OAI	8260	3253	1495	2175	1086	251
OAI train	5782	2277	1047	1522	760	176
OAI val	1239	488	224	326	163	38
OAI test	1239	488	224	327	163	37
External	2295	654	530	380	343	388
External train	1606	458	371	266	240	271
External val	344	98	79	57	52	58
External test	345	98	80	57	51	59

*Note*: The internal osteoarthritis initiative (OAI) counts represent 8260 total images from 4130 unique patients with bilateral knees. The external cohort represents unique patients with one image each (2295 total).

For external evaluation, we used an independent single‐center cohort of 3162 posteroanterior fixed‐flexion knee radiographs acquired between 2020 and 2021 at The Third Affiliated Hospital of Southern Medical University on a Philips “DigitalDiagnost” system. This retrospective deidentified external cohort was approved by the Clinical Research Ethics Committee of The Third Affiliated Hospital of Southern Medical University, with waiver of informed consent; DICOM data were deidentified (including removal of burned‐in identifiers) and analyzed on‐site, whereas OAI data were used as public deidentified data under OAI data‐use terms.

This represented a different acquisition protocol and scanner manufacturer from the OAI cohort, enabling assessment of cross‐site generalization under realistic clinical conditions. Eight musculoskeletal readers followed a two‐stage, cross‐over consensus protocol: in stage 1, the 3162 images were partitioned across readers, with each image graded once; in stage 2, readers received a different, nonoverlapping partition and regraded images they had not seen before, blinded to stage‐1 labels and clinical information. Only images receiving identical KL grades from two independent readers were retained; all others were excluded to ensure high label quality. This consensus process yielded 2295 labeled images from 2295 unique patients (one image per patient). The final grade distribution is displayed in Table 1. Annotators were blinded to any model predictions during the two‐stage grading process, and consensus grading was conducted independently of model development. This cohort has not been previously reported and was specifically collected for this study. Radiographs were deidentified prior to analysis and represent routine clinical acquisitions from 2020 to 2021 on a single scanner and protocol. Inclusion criteria were adult patients (age ≥18 years) with a clinical diagnosis of KOA and a clear radiograph suitable for grading. Exclusion criteria included prior knee arthroplasty/surgery (e.g., total knee arthroplasty or unicompartmental knee arthroplasty) and radiographs of poor quality (blurry/unclear images or prominent artifacts).

### 2.2 Image Preprocessing and Data Augmentation

OAI radiographs were resized to 224 × 224, normalized to ImageNet mean/std, and split at the midline into shared weight left/right halves. External cohort images (≈384 × 384 from DICOM conversion) were deletterboxed by scanning border rows and columns for ≥99.5% near‐black (<8) or near‐white (>247) pixels; any such bars were trimmed on all sides while enforcing at least 50% of the original height and width before resizing to 224 × 224. Each sample comprised the global view plus the two half patches, all fed through the same transform stack.

Geometric and photometric augmentations were applied consistently to global/left/right streams: RandomHorizontalFlip (*p* = 0.5), RandomRotation (±8°), RandomResizedCrop (scale 0.88–1.0, aspect ratio 0.9–1.1), and ColorJitter (brightness/contrast ±0.08), followed by normalization. Test‐time inference used TTA = 8 (identity, horizontal flip, rotations ±3°, ±5°, ±7°) applied identically to all views with probability averaging. All stochastic augmentations were applied only during training; validation and test preprocessing used deterministic resizing and normalization, and test‐time augmentation (TTA) was applied only during inference/evaluation. To justify the selected augmentation budget, we performed a short TTA ablation on the internal validation split comparing TTA = 0/4/8 (Table S1).

### 2.3 Model Architecture

**FIGURE 1:**
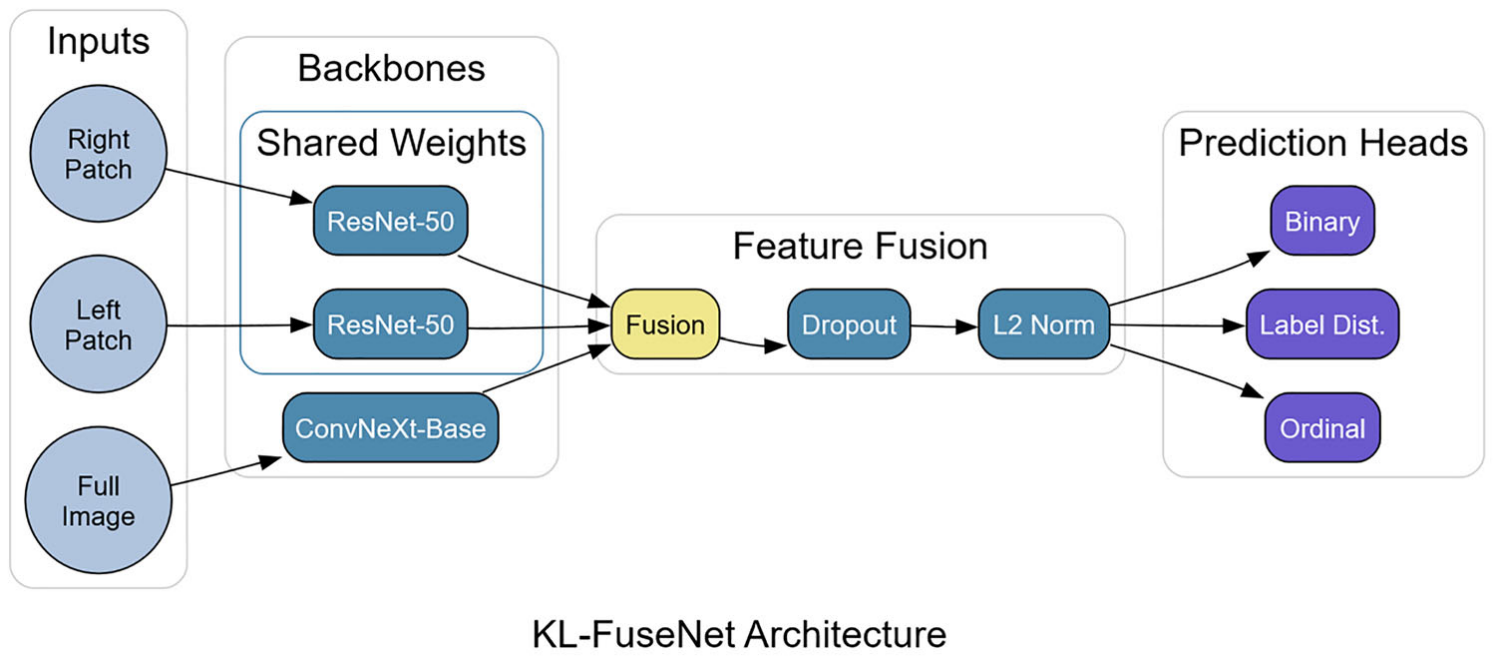
KL‐FuseNet architecture schematic showing the three‐stream design with global (ConvNeXt‐Base) and shared‐weight patch (ResNet‐50) encoders, feature fusion, and three specialized prediction heads.

KL‐FuseNet is a three‐stream, multitask network in PyTorch. The global stream uses ConvNeXt‐Base; the two patch streams share a single ResNet‐50 encoder (Figure 1). Features are obtained via forward_features; if 4‐D, a learnable generalized mean (GeM) pooling layer (*p* initialized to 3.0; *ε* = 1e‐6) adaptively aggregates spatial features, allowing the model to weight informative regions more heavily than standard average pooling [19]; otherwise, we use the feature vector. The three embeddings are concatenated, passed through Dropout (*p* = 0.1), L2‐normalized (torch.nn.functional.normalize), and fed to three linear heads:

1. Ordinal head: four logits trained with binary cross‐entropy (BCE) on cumulative targets 1[*y>k*] for *k* = 0…3 (independent sigmoids). At inference, we enforce nondecreasing thresholds (tuned only on validation) to decode the five‐class grade from ordinal probabilities as in the following equation:
(1) $$\begin{array}{ccc} L_{ord} & = & -\left(\frac{1}{4B}\right)\sum_{i=1}^{B}\sum_{k=1}^{4}\left[t_{\left(ik\right)\log}\left(\sigma \left(o_{ik}\right)\right)+\left(1-t_{ik}\right)\right.\\ & & \left.\log\left(1-\sigma \left(o_{ik}\right)\right)\right],\;where\;\sigma \;\left(x\right)=\frac{1}{1+e^{-x}}\;\;, \end{array}$$

1. Label‑distribution (LD) head: five logits trained with Softmax and KL‑divergence to a Gaussian‑smoothed target (*σ* = 1.0).
2. Binary head: one logit trained with BCE‑with‑logits for KL≥2.

Where *B* is batch size, $o_{ik}$ is the ordinal logit for sample *i* at threshold *k*, $y_{i}$ ∈ {0,1,2,3,4}, and $t_{ik}$ = I($y_{i}$ > *k*‐1) is the cumulative binary target (*I* is the indicator function).

### 2.4 Training Procedure

Optimization used AdamW with two parameter groups (backbones at *LR* = 1e‐4; heads at 2 × *LR*), weight decay 1e‐4, cosine *LR* schedule with three‐epoch warm‐up, mixed precision, and early stopping on validation QWK (patience = 12). We maintained an EMA (decay = 0.999) and saved both raw and EMA checkpoints on improvement.

The composite loss was:

(2) $$L_{total}=\lambda_{ord}\;L_{ord}+\lambda_{ld}L_{ld}+\lambda_{bin}L_{bin},$$

where $L_{total}$ is the total training loss, $L_{ord}$ is the ordinal loss in Equation (1), $L_{ld}$ is the label‐distribution loss (KL divergence between predicted and target KL distributions), and $L_{bin}$ is the binary BCE loss for KL ≥2. The coefficients $\lambda_{ord}$, $\lambda_{ld}$, and $\lambda_{bin}$ are scalar loss weights (set to 1.0, 1.0, and 1.0, respectively, in this configuration). For $L_{ord}$, threshold weights $w_{first}$ and $w_{last}$ were both set to 1.0; for $L_{ld}$, target smoothing used σ=1.0.

Training loss and accuracy were computed as epoch‐level averages over training mini‐batches; validation loss/accuracy and validation QWK were computed on the validation split at the end of each epoch and used for early stopping/model selection. A step‐by‐step pseudocode of the KL‐FuseNet training loop, validation‐based checkpoint selection with early stopping, and TTA‐based inference is provided in Algorithm S1 (Supplementary Materials).

### 2.5 External Fine‐Tuning

Following established protocols for medical imaging domain adaptation [15, 16, 17], we evaluated two transfer scenarios: (1) zero‐shot inference using the OAI‐trained model without modification, and (2) selective fine‐tuning on the external target domain. For the fine‐tuning protocol, we used the independent external cohort of 2295 radiographs with the strict patient‐wise 70/15/15 split described above (1606 training, 344 validation, 345 held‐out test images). The model was initialized from the best OAI checkpoint (KL‑FuseNet) and fine‑tuned exclusively on the external training/validation partitions; the test set remained completely unseen until final evaluation. All parameters were unfrozen (ConvNeXt‑Base global stream, shared‑weight ResNet‑50 patch encoders, and all three prediction heads). We used AdamW with differentiated learning rates (1 × 10^−^^5^ for backbones; 5 × 10^−^^5^ for heads), early stopping patience of 5, and a maximum of 15 epochs, retaining the same composite loss weights (ordinal/label‑distribution/binary) and mixed precision as primary training. Ordinal cutpoints were retuned only on the external validation split (objective: quadratic‑weighted κ), and inference used consistent letterbox handling, 224 × 224 resize/normalization, and TTA = 8. We selected TTA = 8 as a practical performance‐computation trade‐off based on internal development experiments with smaller TTA budgets, which showed a consistent, modest benefit without excessive inference overhead.

### 2.6 Comparative Baselines

We compared against three strong baselines: (1) Flat5C‐ConvNeXt: a flat five‐class classifier using only the global ConvNeXt‐Base stream at 224 × 224; (2) Flat5C‐NFNetF4: a high‐capacity flat five‐class classifier using NFNet‐F4 at 384 × 384; and (3) DualHead‐4C: a multistream model merging KL0 and KL1 grades, using the same global and patch streams as KL‐FuseNet but with simplified dual‐head output. These baselines are also used as targeted ablations of our design: Flat5C‐ConvNeXt removes the patch streams and multitask heads (global‐view, single flat five‐class objective), Flat5C‐NFNetF4 is a higher‐capacity single‐stream baseline, and DualHead‐4C simplifies the output setting by merging KL0/KL1 to test the effect of reduced granularity. Together, they justify the contribution of multistream feature fusion and the ordinal/LD/binary multitask formulation in KL‐FuseNet.

All baselines (Table 2) were trained with the following optimizer, schedule, augmentations, early stopping, and evaluation protocol. All hyperparameter decisions (architecture choice, loss weights, augmentation settings, early stopping/model selection, and TTA) were made using training/validation only; the held‐out test sets were not used for any tuning or selection decisions.

**TABLE 2:** Training configurations for KL grading models.

Model	Classes	Backbone(s)	Image	Batch	Epochs	LR	cosine (warm‐up)
KL‐FuseNet	5	ConvNeXt‐B + Res50	224	32 (x1)	60	1e‐4	cos (3)
DualHead‐ 4C	4	ConvNeXt‐B + Res50	224	32 (x1)	60	1e‐4	cos (3)
Flat5C‐ConvNeXt	5	ConvNeXt‐B	224	32 (x1)	60	1e‐4	cos (3)
Flat5C‐NFNetF4	5	NFNet‐F4	384	8 (x4)	60	5e‐5	cos (6)

*Note*: The table summarizes key hyperparameters for four runs: KL‐FuseNet (dual‐head, 5‐class), DualHead‐4C (4‐class), Flat5C‐ConvNeXt, and Flat5C‐NFNetF4.

### 2.7 Cutpoint Tuning and Inference

After training, ordinal cutpoints were tuned on the validation set only via a lightweight grid/coordinate descent over [0.30–0.70] with a nondecreasing constraint to maximize QWK. Tuned cutpoints were saved and applied unchanged to the corresponding held‐out test sets. No held‐out test set results were used for threshold selection or any other decision; all cutpoints were selected on validation only and then applied unchanged to the corresponding held‐out test sets. Inference used TTA = 8 (identity, hflip, rotations ±5°, ±7°, ±3°) with identical transforms applied to global and patch views, with subsequent logit averaging across augmented samples. For clinically significant OA (KL≥2), this probability was computed as:

(3) $$p\;\left(KL\geq 2\right)=\sum_{c=2}^{4}p\left(c\right),\;$$

where p(c) is the LD‐head predicted probability for class c (c∈{0,1,2,3,4}), and p(KL≥2) is the aggregated probability used for the binary receiver operating characteristic (ROC)/area under the curve (AUC) of clinically significant OA.

### 2.8 Evaluation and Analysis

We report quadratic‐weighted Cohen's kappa (QWK), accuracy, macro‐F1, and mean absolute error (MAE). Accuracy was computed as the proportion of correctly classified images across all samples. MAE was computed as the average absolute difference between predicted and reference KL grades. Macro‐F1 was calculated as the unweighted mean of class‐wise F1 scores, where class‐wise precision and recall were derived from one‐vs‐rest confusion counts. QWK was computed with quadratic disagreement weights across KL grades, comparing observed and chance‐expected agreement. We also provide per‐class precision/recall and F1, confusion matrices, and multiclass ROC/PR curves computed using a one‐vs‐rest formulation; for multiclass ROC, we report the macro‐averaged AUC in figures and tables, with a dashed micro‐average curve overlaid for reference. Binary KL≥2 ROC/PR are computed from p(KL≥2) derived from the label‐distribution head. Patient‐level bootstrap (B = 2000 resamples; patients sampled with replacement with all images per patient retained) is used to obtain 95% confidence intervals for QWK, accuracy, macro‐F1, and MAE on both the OAI internal test and the external held‐out test. For interpretability, we generate Grad‐CAMs by back‐propagating from the label‐distribution head's predicted class through the last convolutional layer (ConvNeXt‐Base and the shared ResNet‐50); heatmaps are normalized to [0,1] and overlaid on 224 × 224 inputs (*alpha* = 0.35), with grids showing representative predicted cases per class from the internal test.

### 2.9 Experimental Setup and Reproducibility

All experiments ran on Windows 11 (build 26100) with Python 3.13.2, PyTorch 2.9.0+cu130 (CUDA 13.0, cuDNN 9.1.2), torchvision 0.24.0+cu130, and torchaudio 2.9.0+cu130. Pretrained backbones came from timm 1.0.20 (ConvNeXt‐Base for the global stream; a weight‐shared ResNet‐50 for both patch streams; NFNet‐F4 for the high‐capacity baseline). Evaluation used scikit‐learn 1.7.2 (quadratic Cohen's kappa for QWK, accuracy, macro‐F1, MAE), NumPy 2.2.6, pandas 2.3.3, and Albumentations 2.0.8; packages were managed with pip 25.3. Training used mixed precision (torch.amp) on a single NVIDIA GeForce RTX 5080 (∼16 GB VRAM; reported 15.92 GB), with a Windows‐safe DataLoader (num_workers = 2). Global/patch encoders were initialized from convnext_base.fb_in22k_ft_in1k.safetensors and resnet50.a1_in1k.safetensors. Reproducibility: seed = 42 across Python/NumPy/PyTorch with cuDNN determinism enabled.

## 3 Results

### 3.1 Overall Performance

**FIGURE 2:**
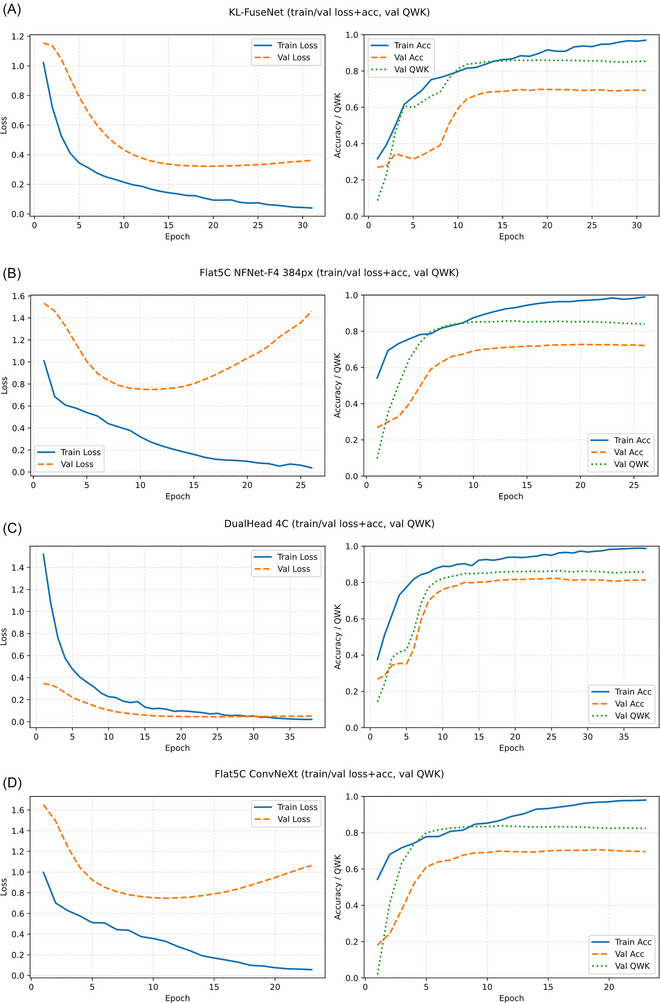
Learning curves for the proposed model and baselines. Each panel contains two subplots: left shows training and validation loss across epochs; right shows training and validation accuracy across epochs, with validation quadratic‑weighted Cohen's kappa (QWK) overlaid. Solid lines denote training, dashed lines denote validation, and the dotted line denotes validation QWK. Panels: (A) KL‑FuseNet, (B) Flat5C‑NFNetF4, (C) DualHead‑4C, (D) Flat5C‑ConvNeXt.

The overall performance of the proposed KL‐FuseNet model and baseline comparators was evaluated on the internal OAI test set (*n* = 1239 images). As summarized in Table 3, the proposed model achieved QWK 0.881 (95% CI 0.866–0.894), accuracy 0.703 (0.678–0.729), macro‐F1 0.724 (0.713–0.766), and MAE 0.313 (0.277–0.331); KL≥2 (AUC 0.981), demonstrating strong ordinal agreement and balanced classification performance. This outperformed the Flat5C‐ConvNeXt (QWK 0.855, accuracy 0.679, macro‐F1 0.701, MAE 0.332) and the higher‐capacity Flat5C‐NFNetF4 (QWK 0.866, accuracy 0.691, macro‐F1 0.735, MAE 0.318) while using lower resolution inputs (224 × 224 vs. 384 × 384 pixels) and fewer parameters. The DualHead‐4C, which merged KL grades 0 and 1 to simplify the task, yielded higher headline metrics (QWK 0.853, accuracy 0.818, macro‐F1 0.803, MAE 0.291) but at the expense of clinical resolution for early stage OA differentiation. Learning curves for all models (Figure 2A−D) illustrate stable convergence, with training performance consistently above validation, and early stopping is driven by validation QWK. We additionally report the final model's train/validation/test performance summary in Table S2 (Supplementary Materials); the held‐out test set was evaluated once at the end, while model selection (including ordinal cutpoints) used the validation split only.

**TABLE 3:** Overall performance metrics on the internal OAI test set across models.

Model	QWK	Accuracy	Macro‐F1	MAE
KL‐FuseNet (proposed)	0.881	0.703	0.724	0.313
Flat5C‐ConvNeXt	0.855	0.679	0.701	0.332
Flat5C‐NFNetF4	0.866	0.691	0.735	0.318
DualHead‐4C (merged KL 0–1)	0.853	0.818	0.803	0.291

**FIGURE 3:**
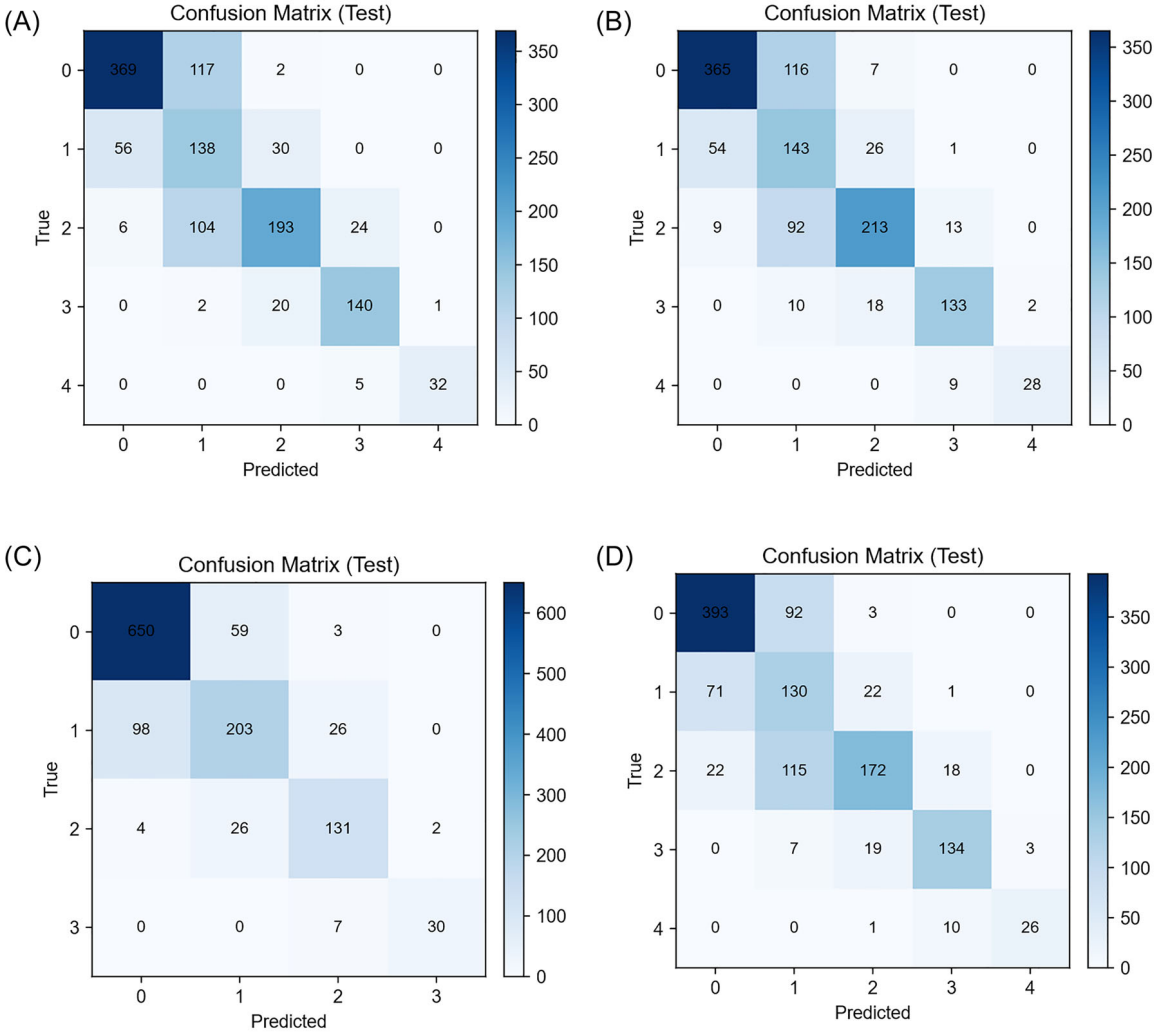
Confusion matrices for all models on the internal OAI test set (*n* = 1239 images), showing row‐normalized classification patterns (values represent proportions; color intensity scales with value). True labels on y‐axis, predicted labels on x‐axis. Panels: (A) KL‐FuseNet, (B) Flat5C‐NFNetF4, (C) DualHead‐4C (*note*: KL0 and KL1 merged), (D) Flat5C‐ConvNeXt.

**FIGURE 4:**
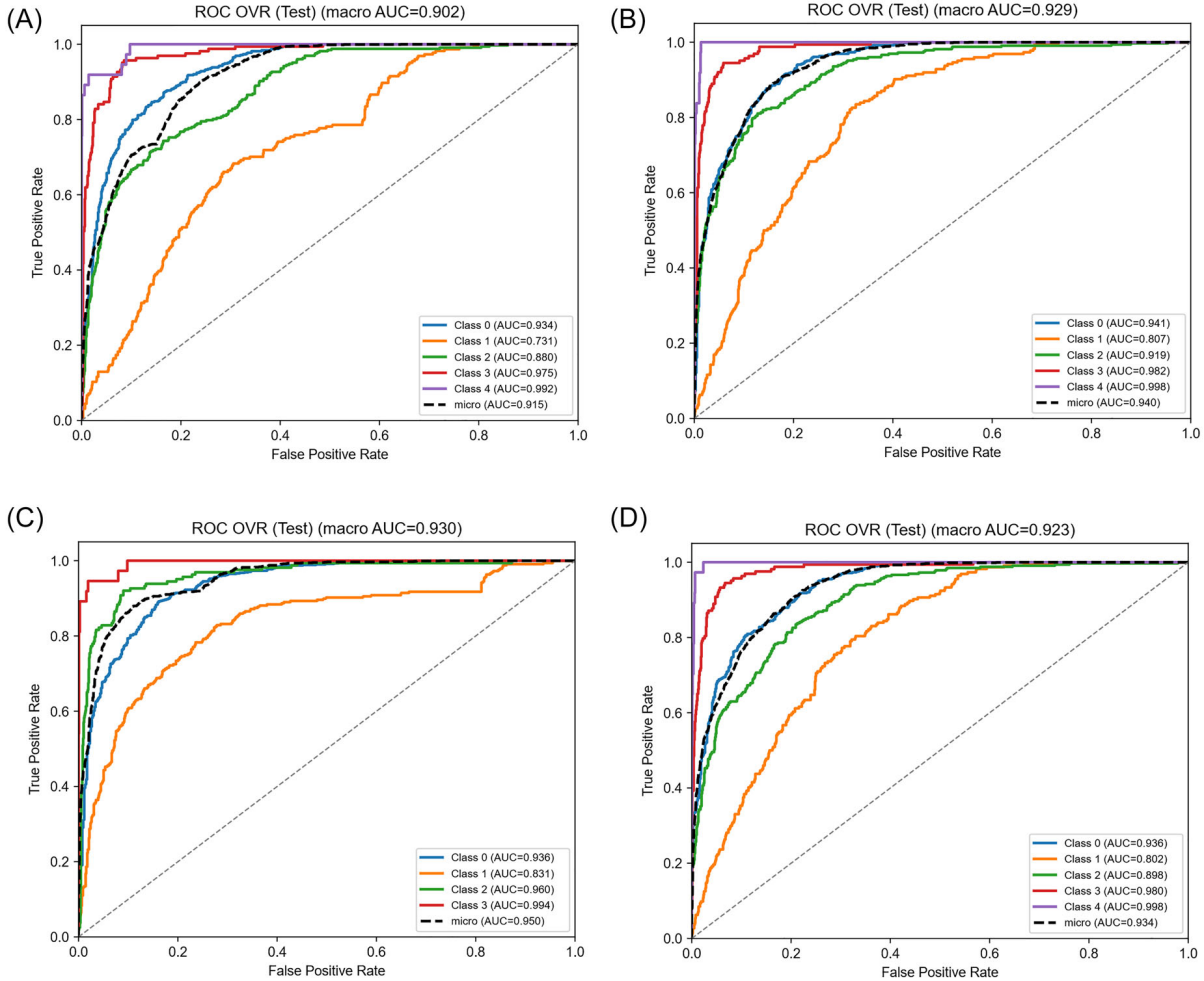
Multiclass receiver operating characteristic (ROC) curves using one‐vs‐rest formulation for all models on the internal OAI test set. Macro‐averaged area under the curve (AUC) reported; dashed line represents micro‐average. Panels: (A) KL‐FuseNet (macro‐AUC = 0.902), (B) Flat5C‐NFNetF4 (macro‐AUC = 0.929), (C) DualHead‐4C (macro‐AUC = 0.930), (D) Flat5C‐ConvNeXt (macro‐AUC = 0.923).

Per‐class metrics on the internal test set show KL‐FuseNet's strength on definitive OA grades: F1  =  0.803 for KL 0 (precision 0.812, recall 0.795), 0.843 for KL 3 (precision 0.796, recall 0.896), and 0.914 for KL 4 (precision 0.944, recall 0.892), reflecting high specificity for non‐OA and end‐stage disease (Table 4). Performance was moderate for KL 2 (F1 0.675, precision 0.788, recall 0.590) and lowest for KL 1 (F1 0.472, precision 0.389, recall 0.616), consistent with the clinical ambiguity of early OA. The KL‐FuseNet confusion matrix in (Figure 3A) shows that 97% of misclassifications are adjacent‐grade errors (e.g., KL 1 predicted as KL 0 or KL 2), which are less critical clinically than nonadjacent confusions. Baseline comparisons underline these differences: Flat5C‐ConvNeXt (Figure 3D) drops to F1 0.458 for KL 1 (precision 0.412, recall 0.527); Flat5C‐NFNetF4 (Figure 3B) raises KL 2 F1 to 0.721 but sacrifices KL 1 precision (0.396, F1 0.489); and the DualHead‐4C (Figure 3C) collapses KL 0/1 into one class, trading early grade specificity for higher aggregate metrics (merged class F1 0.852). Table S3 reports per‐class results for all models, while Figure 4A−D presents multiclass ROC curves and Figure S1 presents the corresponding precision‐recall (PR) curves.

**TABLE 4:** Per‐class metrics for the proposed KL‐FuseNet model on the internal test set.

KL grade	Precision	Recall	F1‐score	Support
0	0.812	0.795	0.803	488
1	0.389	0.616	0.472	224
2	0.788	0.590	0.675	327
3	0.796	0.896	0.843	163
4	0.944	0.892	0.914	37

### 3.2 Ablation Studies

To quantify the contribution of the multistream design under the same leakage‑free patient‑wise split and training protocol, we performed controlled ablations comparing (i) a global‑only model, (ii) a bilateral patch‑only model, and (iii) the full global+patch KL‑FuseNet. This analysis shows that the global stream carries most of the predictive signal, while adding bilateral patch information provides a consistent additional benefit over the global‑only variant. Detailed ablation results are provided in Table S4.

### 3.3 External Cohort Performance

To assess cross‐site generalization and quantify the domain gap from scanner differences, acquisition protocols, and population characteristics, we evaluated KL‐FuseNet under two transfer scenarios on the external held‐out test set (*n* = 345 images).

#### 3.3.1 Zero‐Shot Transfer on Full External Cohort

**FIGURE 5:**
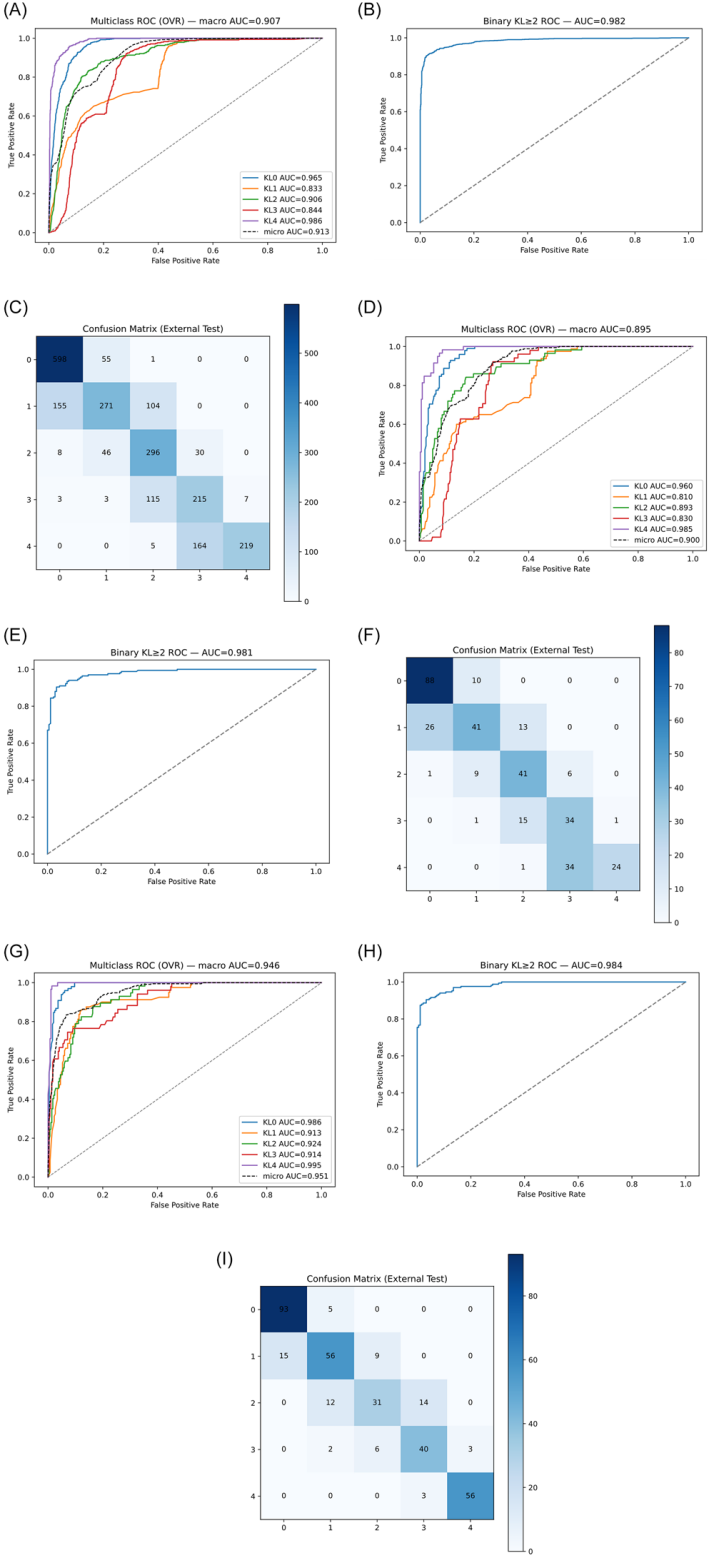
Performance on the external cohort: multiclass ROC curves (A, D, G). Binary KL≥2 ROC curve (B, E, H), and confusion matrix (C, F, I); on zero‐shot transfer on the full external dataset (*n* = 2295), the held‐out test partition (*n* = 345), and fine‐tuned model on the held‐out test partition (*n* = 345), respectively.

Evaluating the OAI‐trained model across the entire external dataset (*n* = 2295) yielded QWK 0.916 (95% CI 0.893–0.920), accuracy 0.697 (0.660–0.710), macro‐F1 0.677 (0.662–0.698), and MAE 0.313 (0.300–0.350); multiclass ROC AUC (macro one‐vs‐rest) was 0.907 and KL≥2 ROC AUC was 0.982 (Figure 5A−C).

#### 3.3.2 Zero‐Shot Transfer on External Held‐Out Test

On the held‐out 15% external test partition (*n* = 345), zero‐shot performance was QWK 0.907 (95% CI 0.869–0.915), accuracy 0.661 (0.605–0.693), macro‐F1 0.634 (0.598–0.692), and MAE 0.348 (0.314–0.402); multiclass ROC AUC was 0.895 and KL≥2 ROC AUC 0.981 (Figure 5D−F). Per‐class analysis showed KL1 F1 0.582, KL0 F1 0.812, and KL4 F1 0.898.

#### 3.3.3 Selective Fine‐Tuning on External Held‐Out Test

**FIGURE 6:**
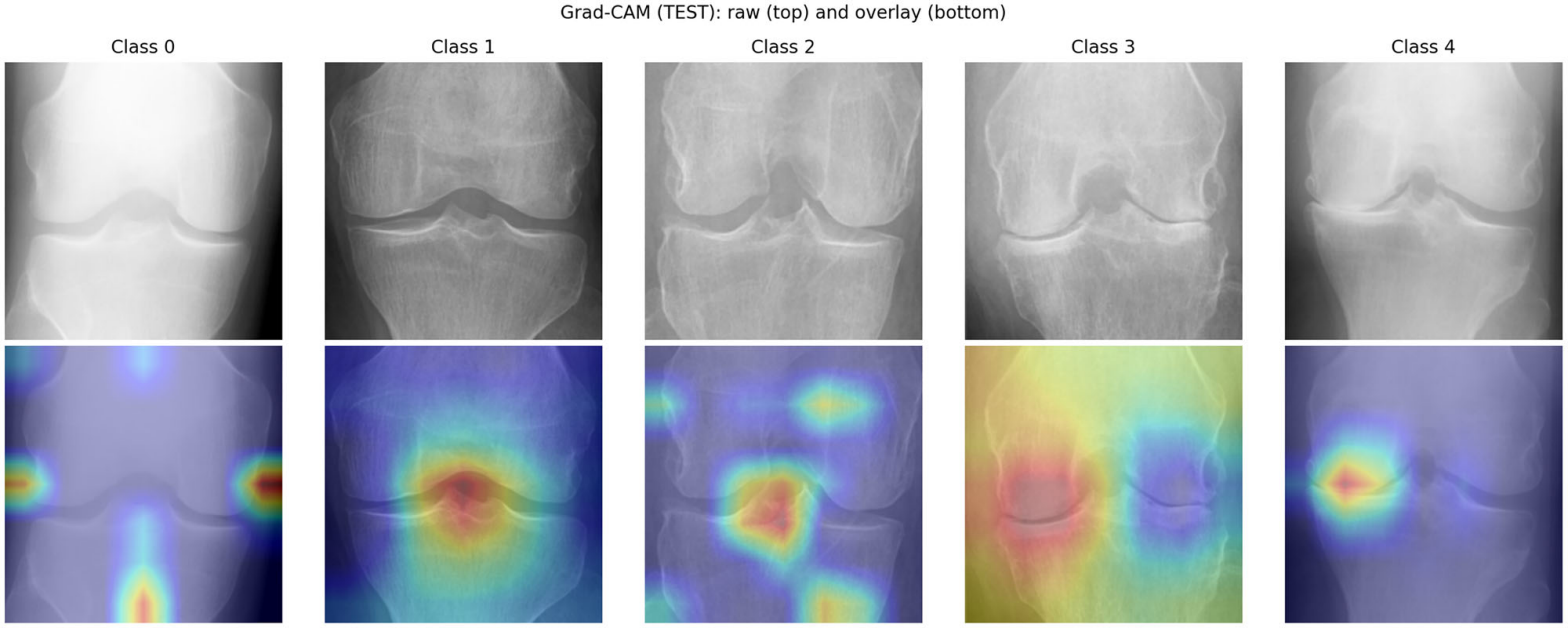
Grad‐CAM visualizations for KL‐FuseNet across all KL grades in internal testing, highlighting attention regions for representative cases.

After fine‐tuning on the external training/validation partitions (*n* = 1606 + 344), the same held‐out test (*n* = 345) achieved QWK 0.950 (0.903–0.960), accuracy 0.800 (0.708–0.831), macro‐F1 0.783 (0.712–0.811), and MAE 0.206 (0.172–0.306); multiclass ROC AUC rose to 0.946 and KL≥2 ROC AUC rose to 0.984 (Figure 6C). Fine‐tuning improved KL1 F1 from 0.582 to 0.723, while definitive grades remained strong (KL0 F1 0.903, KL4 F1 0.949). Confusion matrices (Figure 5G−I) show that misclassifications remained predominantly adjacent‐grade across all scenarios. Complete per‐class metrics appear in Table S3, and Table 5 summarizes aggregate results.

**TABLE 5:** Performance summary of KL‐FuseNet across internal and external evaluation scenarios.

Scenario	*n*	Accuracy (95% CI)	Macro‐F1 (95% CI)	QWK (95% CI)	MAE (95% CI)	KL≥2 ROC AUC	KL≥2 PR AUC
Internal (OAI test)	1239	0.703 (0.678–0.729)	0.724 (0.713–0.766)	0.881 (0.866–0.894)	0.313 (0.277–0.331)	0.981	0.978
External zero‐shot (full cohort)	2295	0.697 (0.660–0.710)	0.677 (0.662–0.698)	0.916 (0.893–0.920)	0.313 (0.300–0.350)	0.982	0.984
External zero‐shot (held‐out test)	345	0.661 (0.605–0.693)	0.634 (0.598–0.692)	0.907 (0.869–0.915)	0.348 (0.314–0.402)	0.981	0.982
External fine‐tuned (held‐out test)	345	0.800 (0.708–0.831)	0.783 (0.712–0.811)	0.950 (0.903–0.960)	0.206 (0.172–0.306)	0.984	0.984

### 3.4 Qualitative Assessment

Grad‐CAM visualizations (Figure 6, columns labeled by class) highlighted attention over tibiofemoral margins, osteophytes, and joint‐space narrowing for higher grades, and global alignment for lower grades.

## 4 Discussion

This study introduced KL‐FuseNet, a multistream, multitask framework for automated KL grading under strict patient‐wise evaluation. The architecture successfully outperformed standard five‐class and higher‐capacity baselines on internal validation, validating its design efficiency.

Our external validation protocol zero‐shot transfer followed by selective fine‐tuning, quantifies the deployment challenge when models encounter different scanners and populations. Here, we use “external validation” to denote evaluation on an independent cohort from a different institution and acquisition setting. Zero‐shot performance (QWK 0.907, accuracy 0.661, macro‐F1 0.634) on the external held‐out test revealed a meaningful domain gap: the 10.7‐point macro‐F1 drop relative to internal validation mirrors cross‐site degradation patterns documented in pneumonia detection [15] and other radiographic tasks [20]. Notably, zero‐shot QWK 0.907 still exceeds most published internal results (Table 6), suggesting inherent cross‐site robustness from our multistream architecture.

**TABLE 6:** Model comparison table for internal performance on rigorous data splits in automated KL grading.

Model	KL‐FuseNet (2025)	Thomas [27]/DenseNet‐169 (2020)	Tiulpin [5]/Siamese CNN (2018)	Pi [12]/Ensemble CNN (2023)	Vaattovaara [28]/DL model (2025)
Validation type	Internal/external (fine‐tuned)	Internal	External	Internal	External
Dataset	OAI (8260; test 1239)/China cohort (2295; test 345)	OAI (40,280 images)	MOST (train, 18,376 images)/OAI (test, 5960 images)	OAI (8260 images)	MOST (train)/OAI (val)
Split type	Patient‐wise	Patient‐wise	Dataset‐wise independent	Image‐level stratified five‐fold CV	Dataset‐wise independent
QWK	0.881/0.950	0.860	0.830	N/A	0.820
Accuracy	0.703/0.800	0.710	0.667	0.769	0.693
MAE	0.313/0.206	N/A	N/A (MSE 0.48)	N/A	N/A
F‐1	0.724/0.783	0.700 (avg)	N/A	0.769	N/A

Selective fine‐tuning on 1950 external images (train + validation) recovered performance substantially: QWK 0.950, accuracy 0.800, macro‐F1 0.783. The 14‐point accuracy and 15‐point macro‐F1 gains demonstrate that modest institution‐specific data can bridge domain gaps, consistent with transfer learning principles [17] and clinical deployment studies [15, 16]. Fine‐tuning particularly benefited KL1 (F1: 0.472 internal → 0.582 zero‐shot → 0.723 fine‐tuned), likely reflecting both domain correction and the external cohort's balanced grade distribution.

The external fine‐tuned QWK (0.950) numerically exceeds internal QWK (0.881), though confidence intervals partially overlap (0.866–0.894 vs. 0.903–0.960). This pattern reflects: (1) smaller external test size (*n* = 345 vs. 1239) with wider uncertainty; (2) more balanced external grade distribution favoring ordinal metrics; (3) systematic differences in acquisition and grading protocols; and (4) targeted optimization for the external distribution. The critical finding is that rigorous fine‐tuning enables cross‐site deployment with performance comparable to or exceeding the training domain, a prerequisite for clinical translation.

Benchmarking requires careful task alignment. Recent studies addressing binary OA screening [21] (custom CNN/ResNet‐50/VGG‐16, 1200 images); [13] (CNN‐based classification, 92.3% binary accuracy) serve complementary clinical roles (presence/absence detection) but are not directly comparable to categorical five‐class KL grading, which requires distinguishing adjacent severity grades. Similarly, studies on knee alignment patterns [22] (16,000 knees, alignment analysis) or continuous anomaly based grading [23], and confidence‐driven early detection [24] address orthogonal diagnostic questions. While these approaches address important complementary questions, our work focuses on the distinct challenge of categorical KL grading. Although the KL scale itself has known limitations, it remains the de facto standard for clinical trials and routine diagnostics, making its robust, reproducible, and generalizable automation a critical and unsolved practical problem. For instance, binary screening studies achieving 92% accuracy [13, 21] often operate under less stringent evaluation (smaller datasets, image‐level splits, simpler binary task) than our five‐class ordinal grading. Our multitask design, by contrast, maintains ordinal structure while still providing strong binary detection (KL≥2 AUC 0.981 internal, 0.984 fine‐tuned). This performance difference reflects task complexity and evaluation rigor, not model weakness.

We note that some prior systems incorporate additional radiographic views (e.g., combined PA+LAT inputs), which can provide extra diagnostic information but also require additional acquisitions compared with single‐view PA‐only protocols. For example, Swiecicki et al. [25] reported strong agreement using a multiview (PA+LAT) deep learning system on MOST with an explicit patient‐level split; by contrast, our primary model is trained on posteroanterior (PA) radiographs only yet performs comparably under strict patient‐wise evaluation. We also note that Li et al. [26] investigated multiview radiographs with prior‐knowledge integration in a single‐center cohort, but the study does not clearly report a strict patient‐wise split and acknowledges correlated (nonindependent) knees, which limits direct performance comparison under leakage‐free patient‐wise evaluation.

Among studies performing categorical KL grading under patient‐wise evaluation, our internal QWK (0.881) matches or exceeds established benchmarks: Thomas et al. (0.86, DenseNet‐169, 40,280 OAI images) [27], Tiulpin et al. (0.83, MOST→OAI transfer) [5], and Vaattovaara et al. (0.82, external MOST) [28]. Our external fine‐tuned QWK (0.950) represents, to our knowledge, one of the highest reported under combined patient‐wise splits and independent external validation. Critically, studies reporting accuracies >75%–80% without patient‐wise splits, such as some ensemble methods [12], likely overestimate performance by 10−20 percentage points through data leakage [8]. Our internal accuracy (70.4%) under strict patient‐wise evaluation provides a more realistic benchmark for generalization. Because cohort selection, grading protocols, preprocessing, and split strategies vary across publications (and some studies use image‐level splits), direct numeric comparisons across papers are inherently limited. To maximize comparability, we prioritized studies that explicitly reported patient‐wise splitting in Table 6.

Recent 2024–2025 work provides additional context for why cross‐study comparisons must be interpreted cautiously, even among papers that aim for rigorous splits. For example, Lee et al. [14] trained on OAI with a patient‐level split and evaluated on an independent MOST cohort, reporting a mean per‐class (grade‐averaged) five‐class accuracy of 75.7% while still showing substantially lower grade‐specific performance for KL1 (43%), underscoring the persistent difficulty of early KOA detection despite architectural enhancements and large‐scale external testing. Pan et al. [29] reported 65.98% test accuracy using an interpretable hierarchical pipeline on Rosenberg‐view radiographs (segmentation/indicator‐driven staged decisions), illustrating a clinically interpretable alternative that introduces additional preprocessing/annotation components and differs from end‐to‐end single‐view workflows. Complementing individual studies, a recent systematic review [30] similarly reported substantially lower sensitivity for KL1 (64.00%) than for severe disease (e.g., KL4 90.32%) and emphasized protocol heterogeneity across studies. Importantly, even the term “accuracy” can be defined differently (e.g., overall accuracy across all images vs. mean per‐class accuracy); therefore, we report overall accuracy alongside macro‐F1 and QWK to better reflect class imbalance and the ordinal nature of KL grading when positioning our results relative to prior work.

Our contribution addresses three persistent gaps: (1) strict patient‐wise evaluation preventing data leakage while maintaining high QWK; (2) quantified cross‐site performance demonstrating 14‐point accuracy improvement (66.1%→80.0%) from zero‐shot to fine‐tuned deployment; and (3) a concrete adaptation protocol for institutional implementation. While individual components (multistream CNNs, ordinal losses, external validation) exist in prior work, their integration under patient‐wise constraints with quantified deployment pathways establishes a reproducible standard for the field.

### 4.1 Ethical Considerations and Public Perspectives

We recognize that clinical deployment of medical AI raises important concerns around algorithmic bias, disparate impact across demographic subgroups, and transparency. While our evaluation includes strict patient‑wise splitting and cross‑institution testing to mitigate common methodological pitfalls, broader assessment of fairness across subgroups and clinical settings will require multisite cohorts with sufficiently rich demographic metadata and prospective evaluation. We, therefore, position this work as decision support rather than an autonomous diagnostic system, and we emphasize the need for transparent reporting, external validation, and ongoing monitoring when models are integrated into real‑world clinical workflows.

### 4.2 Limitations

First, although our external cohort represents an independent clinical site with different equipment and protocols, external validation originates from a single Chinese hospital using Philips DigitalDiagnost. In addition, while we document model development, data provenance/preprocessing, splitting protocols, and external validation in line with established best practices for rigorous medical AI research, prospective multisite studies with predefined analysis plans and ongoing monitoring are needed to support translation to clinical deployment. Federated learning [31] may enable training across heterogeneous sites without data centralization.

Second, KL‐1 performance variation (F1 0.472→0.723) highlights early grade detection challenges. This improvement reflects the external cohort's stricter consensus protocol, balanced distribution, and domain adaptation, but persistent difficulty even for experts reflects genuine clinical ambiguity. Multimodal approaches incorporating clinical variables or magnetic resonance imaging (MRI) biomarkers warrant investigation.

Third, minimum data requirements for effective fine‐tuning remain unclear. Ablation studies varying training size (100−1000 images) would inform deployment with limited labeled data.

Fourth, our evaluation focused on the standard five‐grade KL scale. The multitask design provides a foundation for extending to alternative metrics (continuous scales, anatomical subphenotypes).

Finally, retrospective evaluation on static images requires prospective validation in clinical workflows assessing time‐savings, diagnostic confidence, and clinical decision impact.

## 5 Conclusion

We present KL‐FuseNet, a three‐stream, multitask framework that combines global and patch‐level cues with ordinal, label‐distribution, and binary objectives under rigorous patient‐wise evaluation. By enforcing strict data splits and comprehensive external validation, we provide realistic performance estimates that address pervasive methodological flaws in the literature. The approach delivers strong in‐domain agreement and competitive cross‐site performance, with the significant gap between zero‐shot and fine‐tuned results quantitatively demonstrating the domain adaptation challenge in medical imaging. This framework provides an efficient and interpretable solution for clinical deployment, maintaining full five‐grade granularity while demonstrating the necessity of institution‐specific adaptation for real‐world implementation.

## Author Contributions

T.A. designed the study, collected the data, and drafted the manuscript. A.A. analyzed the data and contributed to the manuscript writing. W.C. helped edit and write the manuscript. H.F. contributed to data curation. Y.L. contributed to data collection and classification. Z.Z. contributed to the overall framework of the study design. R.Z. supervised, planned, and contributed to funding. All authors contributed to manuscript writing and editing. All authors read and approved the final manuscript.

## Funding

This study received no specific funding.

## Conflicts of Interest

The authors declare that they have no conflicts of interest.

## Ethics Approval Statement

This study used two data sources with separate governance. For the external cohort, retrospective use of deidentified radiographs from The Third Affiliated Hospital of Southern Medical University was approved by the Clinical Research Ethics Committee of The Third Affiliated Hospital of Southern Medical University (protocol number not disclosed), with a waiver of informed consent. External DICOM data were deidentified (including removal of burned‐in identifiers) and analyzed on‐site. For OAI, data were obtained as public deidentified data and used under OAI data‐use terms.

## Supporting information




**Supplementary Materials**: nyas70254‐sup‐0001‐SuppMat.docx

## Data Availability

OAI data are available from https://data.mendeley.com/datasets/56rmx5bjcr/1. External cohort data are available from the corresponding author upon reasonable request due to privacy restrictions. Due to an ongoing patent registration process (currently under processing), the exact training and evaluation code can be made available upon reasonable request during peer review, and will be publicly released in a repository and assigned a DOI after completion of the patent process.
