# Supplementary material for: Cross‐Institutional Five‐Class Kellgren–Lawrence Grading of Knee Osteoarthritis via Multitask Deep Learning
Source: Ann N Y Acad Sci. 2026 Mar 14;1557(1):e70254. doi: 10.1111/nyas.70254 (PMC12988769; doi:10.1111/nyas.70254)
Supplement: Supplementary file 1 — Supplementary Materials: nyas70254‐sup‐0001‐SuppMat.docx [file NYAS-1557-0-s001.docx]

**Table S1.** Test-time augmentation (TTA) ablation for KL‑FuseNet on the internal validation split, comparing TTA budgets 0/4/8 (0 = single-view inference without TTA). We report QWK , overall accuracy, macro‑F1, MAE, and measured inference cost in seconds.

| **TTA** | **Loss** | **Accuracy** | **Macro_F1** | **QWK** | **Mae** | **Sec_Total** |
| --- | --- | --- | --- | --- | --- | --- |
| 0 | 0.341 | 0.695 | 0.705 | 0.853 | 0.369 | 10.479 |
| 4 | 0.336 | 0.694 | 0.704 | 0.857 | 0.365 | 10.787 |
| 8 | 0.336 | 0.699 | 0.710 | 0.859 | 0.360 | 16.289 |

**Table S2.** Internal test performance of KL‑FuseNet on the OAI cohort under the leakage‑safe patient‑wise split. The table reports loss, overall accuracy, macro‑F1, QWK, and MAE.

| **Split** | **Loss** | **Accuracy** | **Macro_F1** | **QWK** | **Mae** |
| --- | --- | --- | --- | --- | --- |
| train | 0.188 | 0.896 | 0.888 | 0.946 | 0.142 |
| val | 0.322 | 0.699 | 0.710 | 0.859 | 0.359 |
| test | 0.299 | 0.703 | 0.724 | 0.881 | 0.313 |

**Table. S3.** All models’ metrics per class.

| **Model** | **Cohort** | **Grade** | **Precision** | **Recall** | **F1** | **Support** |
| --- | --- | --- | --- | --- | --- | --- |
| **DualHead-4C** | OAI internal test | 0 | 0.864 | 0.913 | 0.888 | 712 |
| **DualHead-4C** | OAI internal test | 1 | 0.705 | 0.621 | 0.660 | 327 |
| **DualHead-4C** | OAI internal test | 2 | 0.784 | 0.804 | 0.794 | 163 |
| **DualHead-4C** | OAI internal test | 3 | 0.938 | 0.811 | 0.870 | 37 |
| **Flat5C (ConvNeXt-224)** | OAI internal test | 0 | 0.809 | 0.805 | 0.807 | 488 |
| **Flat5C (ConvNeXt-224)** | OAI internal test | 1 | 0.378 | 0.580 | 0.458 | 224 |
| **Flat5C (ConvNeXt-224)** | OAI internal test | 2 | 0.793 | 0.526 | 0.632 | 327 |
| **Flat5C (ConvNeXt-224)** | OAI internal test | 3 | 0.822 | 0.822 | 0.822 | 163 |
| **Flat5C (ConvNeXt-224)** | OAI internal test | 4 | 0.897 | 0.703 | 0.788 | 37 |
| **Flat5C (NFNet-F4 384)** | OAI internal test | 0 | 0.853 | 0.748 | 0.797 | 488 |
| **Flat5C (NFNet-F4 384)** | OAI internal test | 1 | 0.396 | 0.638 | 0.489 | 224 |
| **Flat5C (NFNet-F4 384)** | OAI internal test | 2 | 0.807 | 0.651 | 0.721 | 327 |
| **Flat5C (NFNet-F4 384)** | OAI internal test | 3 | 0.853 | 0.816 | 0.834 | 163 |
| **Flat5C (NFNet-F4 384)** | OAI internal test | 4 | 0.933 | 0.757 | 0.836 | 37 |
| **KL-FuseNet** | OAI internal test | 0 | 0.856 | 0.756 | 0.803 | 488 |
| **KL-FuseNet** | OAI internal test | 1 | 0.382 | 0.616 | 0.472 | 224 |
| **KL-FuseNet** | OAI internal test | 2 | 0.788 | 0.590 | 0.675 | 327 |
| **KL-FuseNet** | OAI internal test | 3 | 0.828 | 0.859 | 0.843 | 163 |
| **KL-FuseNet** | OAI internal test | 4 | 0.970 | 0.865 | 0.914 | 37 |
| **KL-FuseNet** | External Baseline (all) | 0 | 0.783 | 0.914 | 0.843 | 654 |
| **KL-FuseNet** | External Baseline (all) | 1 | 0.723 | 0.511 | 0.599 | 530 |
| **KL-FuseNet** | External Baseline (all) | 2 | 0.568 | 0.779 | 0.657 | 380 |
| **KL-FuseNet** | External Baseline (all) | 3 | 0.526 | 0.627 | 0.572 | 343 |
| **KL-FuseNet** | External Baseline (all) | 4 | 0.969 | 0.564 | 0.713 | 388 |
| **KL-FuseNet** | External Baseline (70/15/15) | 0 | 0.765 | 0.898 | 0.826 | 98 |
| **KL-FuseNet** | External Baseline (70/15/15) | 1 | 0.672 | 0.513 | 0.582 | 80 |
| **KL-FuseNet** | External Baseline (70/15/15) | 2 | 0.586 | 0.719 | 0.646 | 57 |
| **KL-FuseNet** | External Baseline (70/15/15) | 3 | 0.459 | 0.667 | 0.544 | 51 |
| **KL-FuseNet** | External Baseline (70/15/15) | 4 | 0.960 | 0.407 | 0.571 | 59 |
| **KL-FuseNet** | External Fine-tuned (70/15/15) | 0 | 0.861 | 0.949 | 0.903 | 98 |
| **KL-FuseNet** | External Fine-tuned (70/15/15) | 1 | 0.747 | 0.700 | 0.723 | 80 |
| **KL-FuseNet** | External Fine-tuned (70/15/15) | 2 | 0.674 | 0.544 | 0.602 | 57 |
| **KL-FuseNet** | External Fine-tuned (70/15/15) | 3 | 0.702 | 0.784 | 0.741 | 51 |
| **KL-FuseNet** | External Fine-tuned (70/15/15) | 4 | 0.949 | 0.949 | 0.949 | 59 |

**Table S4.** Controlled architecture ablation study under the same leakage‑safe patient‑wise split and training protocol. We report internal test performance (QWK, overall accuracy, macro‑F1, and MAE) for KL‑FuseNet (global+patch) and the corresponding ablated variants (global‑only and patch‑only).

| **Model** | **Test QWK** | **Test accuracy** | **Test macro_f1** | **Test mae** |
| --- | --- | --- | --- | --- |
| KL-FuseNet (global+patch) | 0.881 | 0.703 | 0.724 | 0.313 |
| Global-only | 0.875 | 0.692 | 0.723 | 0.322 |
| Patch-only | 0.555 | 0.397 | 0.419 | 0.822 |

**Figure. S1.** ROC/PR curves for KL-FuseNet and other baselines.

| 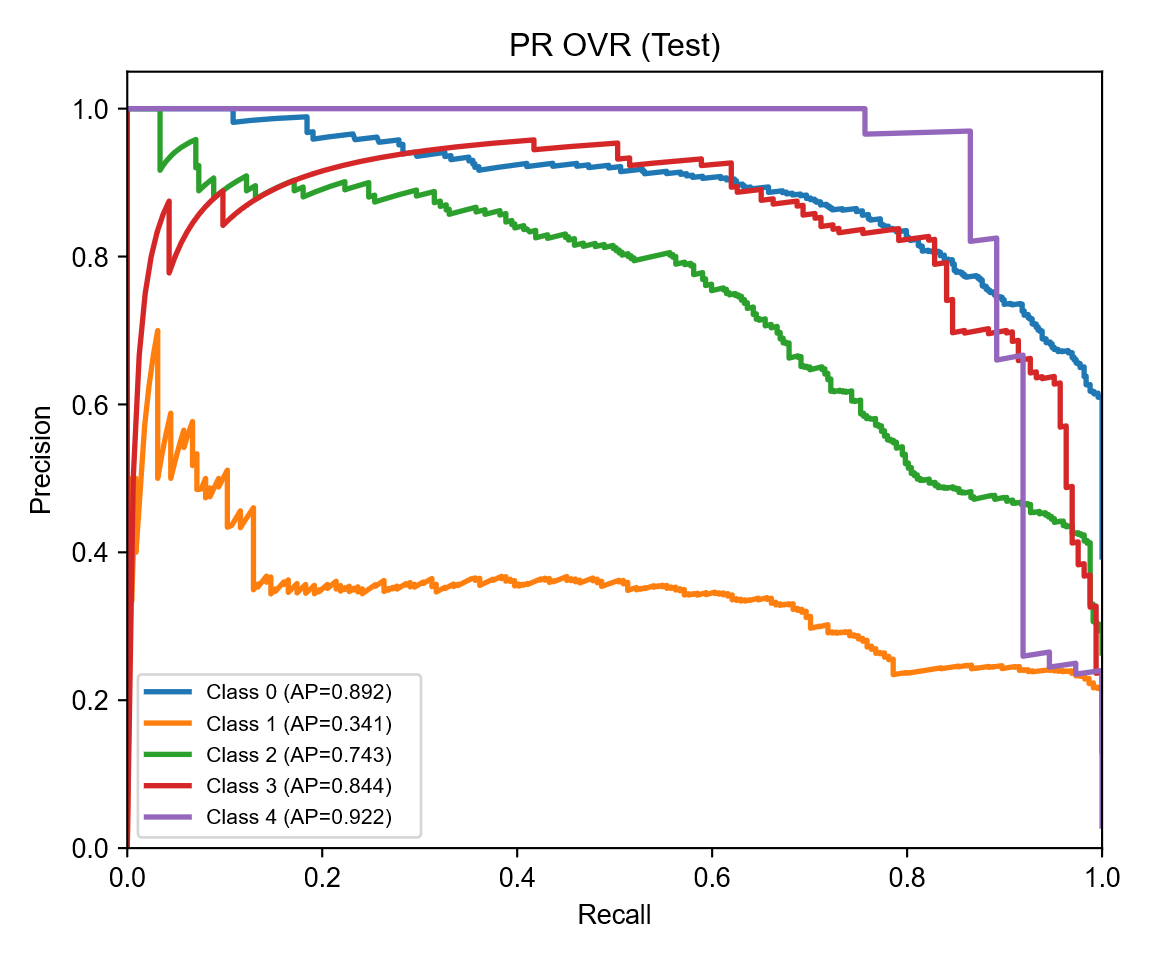 | 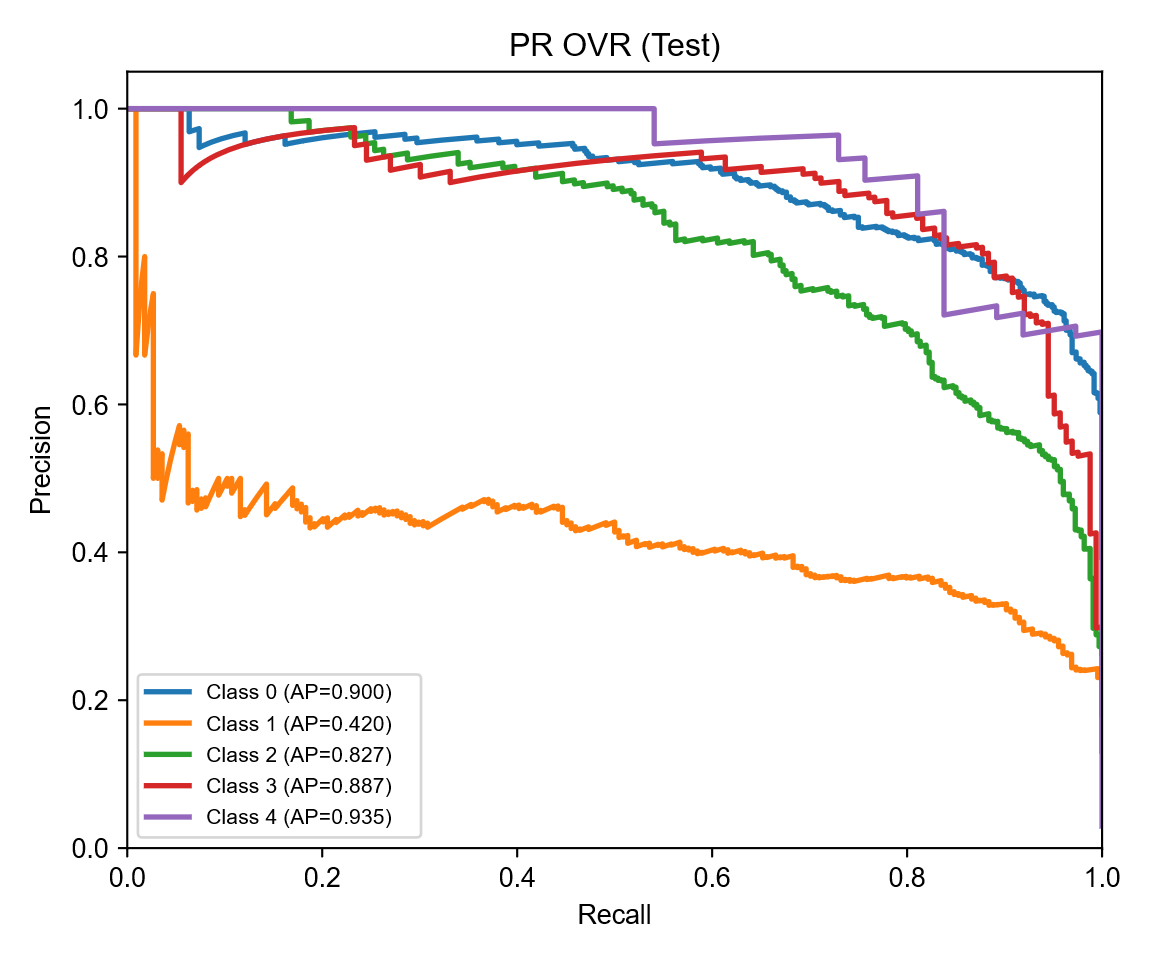 |
| --- | --- |
| **(A) KL-FuseNet** | **(B) Flat5C-NFNetF4** |
| 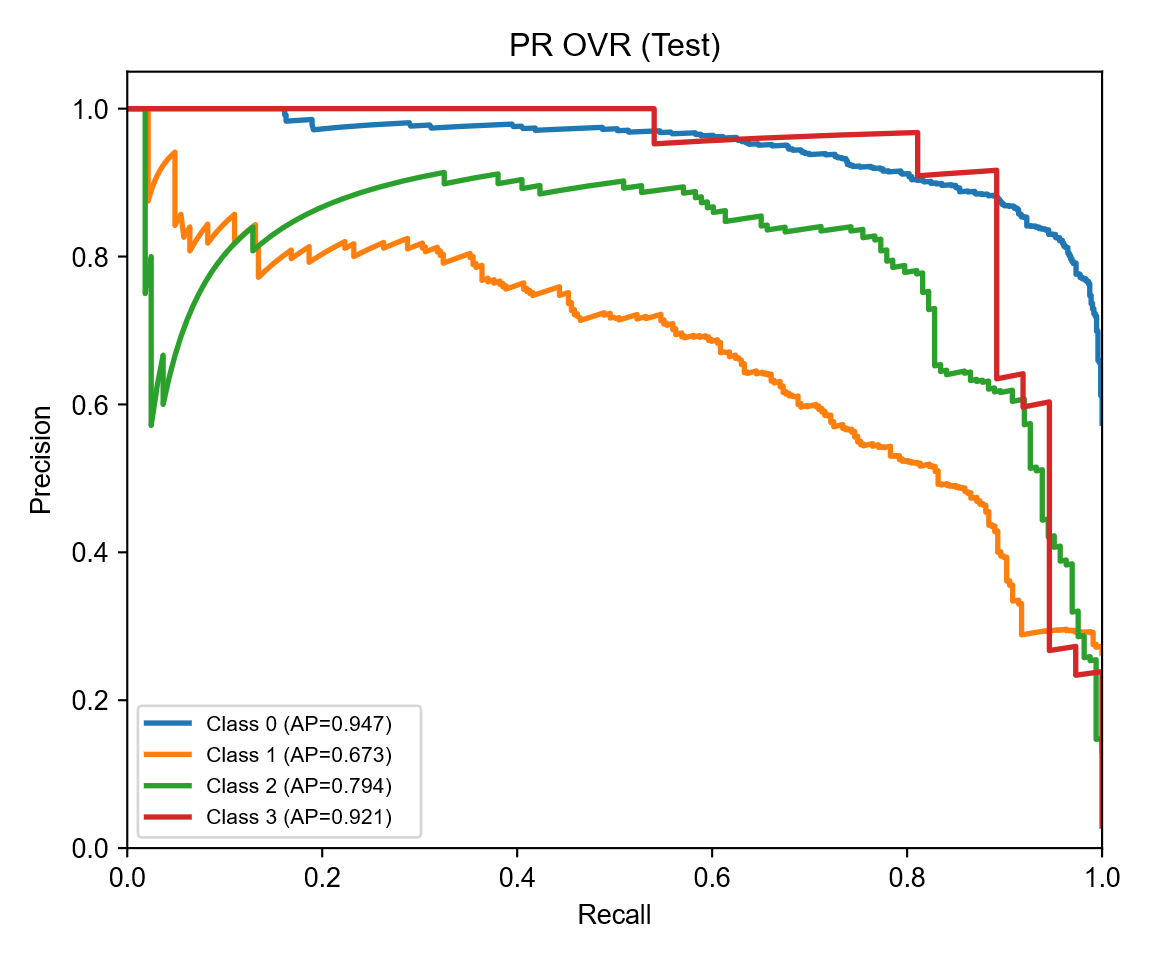 | 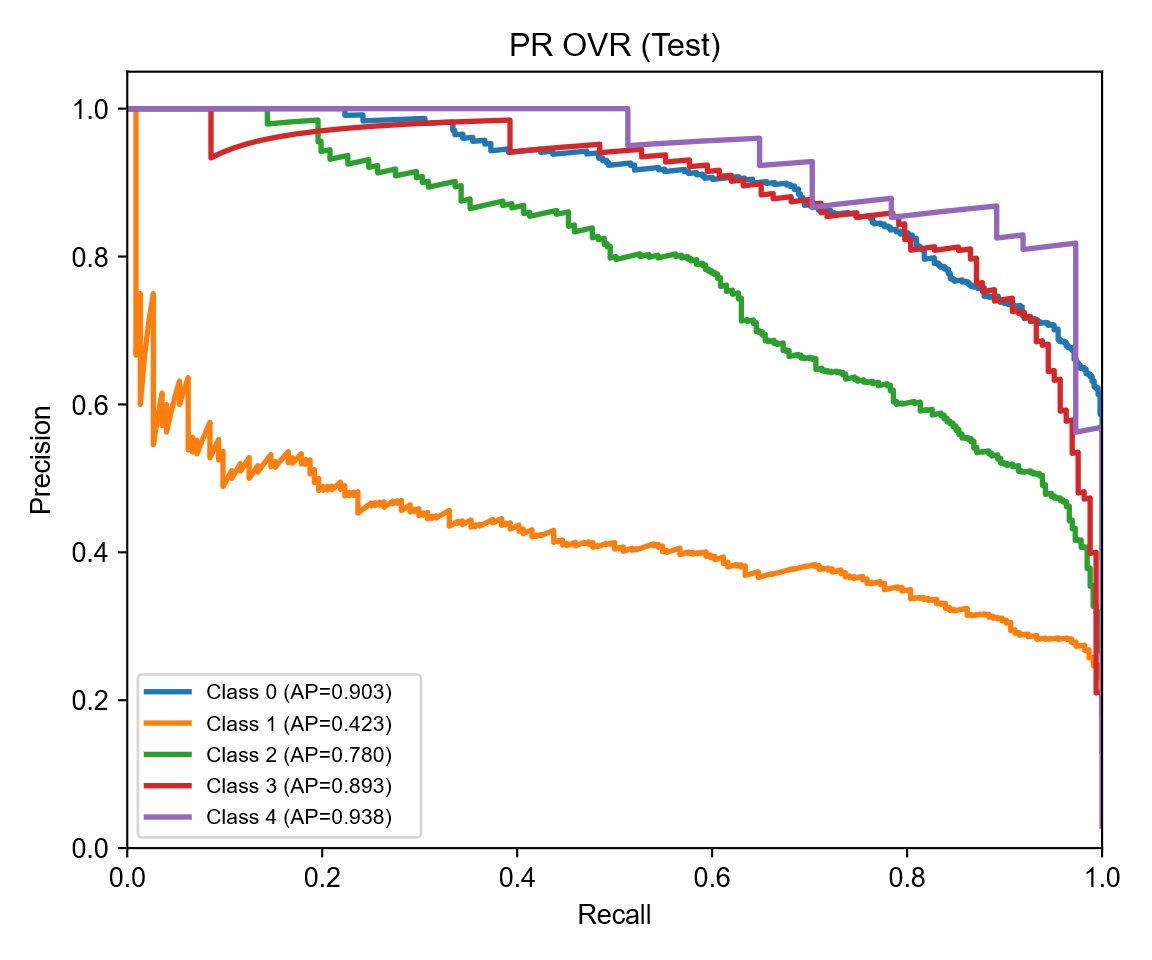 |
| **(C) DualHead-4C** | **(D) Flat5C-ConvNeXt** |

**Algorithm S1.** KL-FuseNet Training and Inference Pseudocode. This pseudocode summarizes the high-level training, validation, early stopping, and TTA inference workflow used in the KL-FuseNet pipeline under leakage-safe patient-wise splitting.

Inputs:

D_train, D_val, D_test (patient-wise split)

Hyperparameters: epochs E, batch size B, patience P, learning rates,

loss weights (lambda_ord, lambda_ld, lambda_bin),

edge weights (thr_w_first, thr_w_last), TTA views K=8

Model:

Global stream: ConvNeXt-Base

Patch streams: left and right patches with shared ResNet-50 weights

Stream pooling: GeM on each stream

Fusion: z = L2Norm(Dropout(Concat(f_global, f_left, f_right)))

Heads: ordinal head (4 logits), label-distribution head (5 logits),

binary head (KL>=2)

Procedure FORWARD(full, left, right):

f_global <- GeM(ConvNeXt.forward_features(full))

f_left <- GeM(ResNet.forward_features(left))

f_right <- GeM(ResNet.forward_features(right))

z <- L2Norm(Dropout(Concat(f_global, f_left, f_right)))

o <- OrdinalHead(z); d <- LDHead(z); b <- BinaryHead(z)

return (o, d, b)

Procedure TRAIN():

Initialize optimizer (backbone and head parameter groups)

Initialize mixed-precision scaler and optional EMA

best_qwk <- -inf; no_improve <- 0

for epoch = 1..E:

for each batch in D_train:

(o, d, b) <- FORWARD(full, left, right)

t_ord[k] <- 1[y > k], k=0..3

t_ld <- smoothed 5-class label distribution

t_bin <- 1[y >= 2]

L_ord <- weighted BCEWithLogits(o, t_ord)

with edge weights on first and last ordinal terms

L_ld <- KLDiv(log_softmax(d), t_ld)

L_bin <- BCEWithLogits(b, t_bin)

L_total <- lambda_ord*L_ord + lambda_ld*L_ld + lambda_bin*L_bin

Backpropagate L_total, update optimizer and scaler

If EMA enabled: update EMA parameters

qwk_val <- EVALUATE(D_val, thresholds=None, TTA=K).QWK

if qwk_val > best_qwk:

best_qwk <- qwk_val; save best checkpoint; no_improve <- 0

else:

no_improve <- no_improve + 1

if no_improve >= P:

stop training

Procedure TUNE_CUTPOINTS_ON_VAL():

Load best checkpoint

Collect ordinal probabilities on D_val (TTA=K)

Search thresholds tau0..tau3 on validation set

Enforce non-decreasing order tau0 <= tau1 <= tau2 <= tau3

Select thresholds maximizing validation QWK

Save tuned thresholds

Procedure FORWARD_TTA(full, left, right, K):

if K=8, use: identity, hflip, rot(-5), rot(+5), rot(-7), rot(+7), rot(-3), rot(+3)

for each transform i in K views:

(o_i, d_i, b_i) <- FORWARD(transform_i(full, left, right))

return averaged logits: o_bar, d_bar, b_bar

Procedure INFER(image):

Split image into full, left patch, right patch

(o_bar, d_bar, b_bar) <- FORWARD_TTA(full, left, right, K=8)

p_ord <- sigmoid(o_bar)

grade_hat <- sum_k 1[p_ord[k] > tau_k] using tuned validation thresholds

p_ge2 <- sum_{c=2..4} softmax(d_bar)[c]

return grade_hat, p_ge2, softmax(d_bar), p_ord

Main:

TRAIN()

TUNE_CUTPOINTS_ON_VAL()

Evaluate once on D_test with tuned thresholds and TTA=8
